# Effect of sodium butyrate on HDAC8 mRNA expression in colorectal cancer cell lines and molecular docking study of LHX1 - sodium butyrate interaction

**DOI:** 10.17179/excli2020-2010

**Published:** 2020-07-23

**Authors:** Flora Forouzesh, Mahsa Ghiaghi, Hamzeh Rahimi

**Affiliations:** 1 Department of Genetics, Faculty of Advanced Science and Technology, Tehran Medical Sciences, Islamic Azad University, Tehran, Iran; 2 Department of Molecular Medicine, Pasteur Institute of Iran, Tehran, Iran

**Keywords:** colorectal cancer, sodium butyrate, HDAC8 gene, LHX1 transcription factor, docking, HT-29, HCT-116 cell lines

## Abstract

Colorectal cancer (CRC) is the third most common type of cancer and the fourth leading cause of cancer related deaths worldwide. The Histone Deacetylase 8 (HDAC8) gene is a gene with unique features which can be used as a potential target for drug design. The LHX1 transcription factor is an important transcription factor for this gene. The aim of this study was to investigate the effect of sodium butyrate (NaB) as a histone deacetylase inhibitor (HDACi) on the expression of the HDAC8 gene in the colorectal cancer cell line, and the molecular docking of the LHX1 transcription factor with NaB. For this purpose, HCT-116 and HT-29 cell lines were treated with different concentrations of NaB (6.25 mM to 150 mM) at 24, 48 and 72 hours. Subsequently, RNA was extracted from the treated and untreated cells and cDNA was synthesized. Quantitative Real-Time-PCR was done to investigate the mRNA expression of HDAC8. Molecular docking was also performed to investigate the interaction between NaB and LHX1. Based on Real-time-PCR results, the concentration of 150 mM of NaB after 24 hours in HT-29 and HCT-116 cell lines caused a significant reduction in mRNA expression of HDAC8 (P<0.05). After 48 hours of treatment, there was a significant decrease in the mRNA expression of HDAC8 at all concentrations (P<0.05). The docking results showed that LHX1 and NaB interacted best at the lowest energy levels. Our results also showed that NaB bonded strongly to LHX1. In addition, our results demonstrated that NaB bound to the LHX1 transcription factor and inhibited the function of this factor and consequently decreased the transcription from the HDAC8 gene which resulted in cell death. Future studies are needed to assess the likely molecular mechanisms of NaB action on gene expression.

## Introduction

Colorectal cancer (CRC) is the fourth most common cause of death from cancer in the world (Ansari et al., 2006[2]; Mundade et al., 2014[34]; Stigliano et al., 2014[44]). As it has been found, cancer is the result of a wide range of genetic and epigenetic changes. Histone acetylation is one of such epigenetic changes (Esteller, 2002[11]; Johnstone, 2002[23]; Iizuka and Smith, 2003[22]). Acetylation levels are the result of the balance of the activities between histone deacetylase (HDAC) enzymes and histone acetyltransferase (HAT) enzymes (Bannister and Kouzarides, 2011[3]). The HDAC8 gene is a class I HDAC gene (Hu et al., 2000[21]; Van den Wyngaert et al., 2000[48]; Wawruszak et al., 2019[53]), which is present in the nucleus and cytoplasm of cells distinctively (Waltregny et al., 2004[52]; Li et al., 2014[28]). HDAC8 is expressed in normal tissues, but its expression increases in cancerous tissues (Song et al., 2015[43]). For example, HDAC8 is correlated with T-cell lymphoma, childhood neuroblastoma and gastric, colon, lung, breast cancers. In addition, HDAC8 shows functional abundance with HDAC6 when overexpressed in Hela and cervical cancer cells (Vanaja et al., 2018[49]). As a result, HDAC8 can potentially be an attractive target for structure-based drug design because it exhibits a number of unique characteristics allowing the development of selective inhibitors. One particularly interesting feature in this regard is the formation of the foot pocket also called the acetate release channel, which is a sub-pocket observed in class I HDACs, such as HDAC1, 2, 3 and 8, but not in class II isotypes such as, HDAC4 and 7 X-ray structures (Hildmann et al., 2007[19]). HDACs are regulators of gene expression, that affect cell cycle arrest, apoptosis, angiogenesis, and the differentiation of different cell types (Hildmann et al., 2007[19]; Riester et al., 2007[38]). Moreover, abnormal histone modification patterns have been found to be closely associated with several diseases such as cancers (Ma et al., 2019[30]). According to the Human Protein Atlas (2019[47]), these types of class I HDACs are expressed in several types of tumors. The pharmacological inhibition of HDACs also known as HDACi may be considered as new method for cancer therapy (Bolden et al., 2006[7]; Minucci and Pelicci, 2006[32]; Hildmann et al., 2007[19]; Riester et al., 2007[38]; Shuttleworth et al., 2010[40]). It has been observed that, HDACi can change the balance between HATs and HDACs and therefore lead to a hyperacetylated state of histones that causes cell cycle arrest, inducing transcriptional inhibition and apoptosis which are valid strategies for cancer treatment (Bolden et al., 2006[7]). 

One of these HDACis is sodium butyrate (NaB). Butyrate is produced by anaerobic bacterial fermentation of dietary fibers and is a short-chain fatty acid (Emenaker et al., 2001[10]; Hinnebusch et al., 2002[20]). One of the functions of butyrate is the anti-inflammatory effect that plays a role in inhibiting histone deacetylase. Butyrate specifically inhibits the class I HDAC. And it has the highest level of inhibition for HDAC8 (Cummings and Macfarlane, 1991[9]). Hence, the production of butyrate in the colon may protect against colon cancer and inhibit the development of colon cancer (Emenaker et al., 2001[10]; He et al., 2001[18]). As a result of such findings, identifying the chemical properties that govern the action of NaB on HDAC8 could be significant for the development of new selective drugs with low side effects. It is also known that logical drug design involves investigating new molecules that complement the molecular target in structure, aiming to reach an allosteric site or the active site (Bieliauskas and Pflum, 2008[6]; Estiu et al., 2008[12], 2010[13]; Nussinov and Tsai, 2012[36]). Moreover, molecular docking can be defined as a simulation process where a ligand position is estimated at a predicted or pre-defined binding site. This process can be used widely; with minimal effort and expense, and it may increase the effectiveness of obtaining potential drugs. Through molecular docking, we aim to clarify the beneficial HDAC8 structural features which may help to obtain knowledge for drug development in the future using some known selective HDACis (Knegtel et al., 1997[26]; Alonso et al., 2006[1]).

The LHX1 protein is one of the transcription factors involved in the expression of the HDAC8 gene. The LIM homeobox 1 protein of the human cell is encoded by the LHX1 gene. This protein is a transcription factor, important for control of the development and differentiation of neural, lymphoid, renal and urogenital cells. The LHX protein plays a role in determining the origin and identity of the cell. In humans, the encoding genome is located on chromosome 17q11.2-q12. Its molecular weight is 44.808 Dalton and it contains 406 amino acids. LHX1 binds to DNA through its homeodomains and is an important evolutionary regulator. The LHX1 protein contains 3 domains, which include: A homeobox domain for DNA binding, and two LIM domains, which are involved in the ion-zinc connection. The LIM domains involved in zinc ion interconnection, play a role in cytoskeleton organization, cell adhesion, epithelial development, gene transcription, mRNA trafficking, translation, protein folding, and chromatin remodeling. In addition, LIM domains have seven conserved cysteine residues and a histidine molecule. LIM domains bind two zinc ions. However, LIM does not bind DNA, rather it seems to act as a junction for protein-protein interaction (Phillips, 2002[37]; Karavanov et al., 1998[24]; Kobayashi et al., 2004[27]; Ye et al., 2012[56]). 

Additionally, studies showed that sodium butyrate affected the expression of genes through binding to transcription factors. It was found in previous studies that, HDAC8 regulated the transcription factors of LHX1 and Otx2 through histone H3 hyperacetylation in their promoter regions (Saha et al., 2013[39]).

Therefore, in this study, we evaluated the effects of NaB on mRNA expression of the HDAC8 gene in human colorectal cancer cell lines and also how the drug affects gene expression. It was important to identify the molecular mechanism of NaB action on gene expression. In addition a molecular Docking study between NaB and LHX1 as a transcription factor of HDAC8 was done in this study.

## Materials and Methods

### Cell culture

HCT-116 (the human colorectal carcinoma) and HT-29 (the colorectal adenocarcinoma) cell lines were obtained from the Iranian Biological Research Center (Pastor, Tehran, Iran). HCT-116 and HT-29 cell lines were cultured in Dulbecco's modified Eagle's medium (DMEM) and RPMI 1640, respectively, ( Gibco by life technologies, Germany) including 10 % fetal bovine serum (FBS; Gibco by life technologies, Germany) and 1 % penicillin/streptomycin (Dacell, Iran) at 37 °C and 5 % CO_2_. The cells were grown to approximately 80 % confluence. 

### Cell treatment and cytotoxicity assay 

CRC cell lines were plated at 50×10^3^ cells per well in a 200 μL volume in 96-well plates. CRC cell lines were treated with the medium containing 6.25 to 200 mM of NaB, (Biobasic, Canada Inc.) according to our previous study, (Ghiaghi et al., 2019[15]) for 24 and 48 hours. Untreated cells (0 Mm) were used as negative control. Cell viability was determined by 3-(4,5-dimethylthiazol-2-yl)-2,5-diphenyltetrazolium bromide (MTT) assay according to our previous paper (Ghiaghi et al., 2019[15]). All experiments were designed in triplicates.

### RNA extraction and cDNA synthesis

Total RNA was isolated from untreated CRC cell lines and NaB-treated cells using RNX-plus solutions (Sinaclon, Iran) by following the manufacturer’s recommendations. Quality and quantity control of RNA purity was determined by agarose gel electrophoresis and OD 260/280 ratio measurements by Biophotometer plus, (Eppendorf, Hamburg, Germany) respectively. 2000 ng of RNA was reverse-transcribed to cDNA using the Yektatajhiz Kit (Cat. No. YT4500, Iran), according to the manufacturer's protocol.

### Quantitative Real-Time PCR (qRT-PCR)

The mRNA expression of HDAC8 was assayed by Real-Time PCR. In the assay, SYBR Greene was used as a reporter dye. Real-Time PCR reactions were performed via system Light Cycler 96, (Roche Diagnostics, Germany). Samples were amplified in duplicates by RT-PCR. To evaluate the data, the 2−ΔΔCtapproximation was utilized with GAPDH as an internal reference gene. Primer (10 pmol) sequences for qRT-PCR are listed in Table 1(Tab. 1).

### Protein structure prediction

The FASTA form of the LHX1 introduced on the Interproscan site was used to obtain all necessary information for the LHX1 domains. For this purpose, the sequence of LHX1 protein was downloaded from the UniProt database (www.uniprot.org). Then structure-sequence alignment was performed using HHpred against the PDB database to find the best templates for homology modeling. Additionally, coordinates of LHX1 protein structures were downloaded from the Protein Data Bank (PDB) (www.rcsb.org). The Modeller software (version 17) program (http://www.salilab.org) (Marti-Renom et al., 2000[31]; Webb and Sali, 2016[54]) and the PDB (Berman et al., 2003[5]) of LHX1 molecule were used as a template to simulate and extract a model with the energy of all the atoms in the protein amino acid sequences to have LHX1 action. For this purpose, firstly the amino acid sequence was entered into the I-tasser server, and I-tasser provided the protein structure for us. Then the I-tasser model to the Galaxy server was refined, which operated according to a common refinement method. This method initially reconstructed the lateral chains and then, using molecular dynamics simulation, predicted the chain dispersion and relaxation of the overall structure of the protein. The best model was chosen from the Galaxy server to refine the server, and the resulting model was then introduced into the Modeller software to construct 1000 models with different energy levels for the LHX1 amino acid sequence forming atoms. Subsequently, the model that had the lowest energy level and DOPE Score for the atoms was chosen as the most preferable model using the VADAR and PSVS servers. These servers provided a concise confirmation report, which included a standard set of charts and tables, which were tables and charts related to Ramachandran, Verify3D (https://servicesn.mbi.ucla.edu/Verify3D) (Luthy et al., 1992[29]), and MolProbity (http://molprobity.biochem.duke.edu/), which were used to analyze the data. In this way, the best model for the LHX1 came with the desired tables and charts. Then, the Pymol software (version 1.7.4.5 Edu) (www.pymol.org) and Chimera software (version 1.11.2) (http://www.cgl.ucsf.edu/chimera/) were used to display the tertiary structure (3D) of the LHX1 protein. The final structure energy was minimized in GROMACS (version 5) (http://www.gromacs.org) (Berendsen et al., 1995[4]).

### Molecular docking and molecular interaction studies

After modeling and obtaining a three-dimensional (3D) protein structure, we had to perform a docking between LHX1 and sodium butyrate. The LHX1 protein FASTA form was introduced on the sites of the 3D ligand site and the Uchicago.edu/binding site to determine the location of the amino acid binding sites. After the investigation, the best linking sites were identified with their related amino acids. We used the Autodock vina software (version 4.2.6) (http://vina.scripps.edu/) (Morris et al., 2009[33]) to identify the docking links obtained from the binding of LHX1 with sodium butyrate. In the Autodock Tools, the receptor and ligand preparation included; removal of all water molecules from protein, adjustment of atom types, adding the charge and adding polar hydrogen atoms. Using Autogrid as part of the Autodock software, sodium butyrate was placed in a three-dimensional grid sized x,y,z. The lowest energy level was selected and used as the most suitable binding method, which could be used to evaluate the binding of LHX1 protein and sodium butyrate ligand in Pymol software. Finally, the Ligplot software (version 4.5.3) (www.ebi.ac.uk) (Wallace et al., 1995[51]) was used to generate a two-dimensional (2D) schematic of the protein-ligand complex. In this application, a mixture of intermolecular interactions including hydrogen bonds, water interactions, etc. between LHX1 and NaB, could be observed.

### Statistical analysis 

Each treatment was performed in triplicates and each test was repeated at least twice. Statistical analysis was undertaken using SPSS 17.0 and the One-way ANOVA method. The results shown, are representative of three independent experiments. P-value <0.05 was considered to be statistically significant. Data analysis of the Docking section was done with the help of the Molprobity, Ramachandran, Verify3D, Autodock vina and the Ligplot software.

## Results

### Effect of NaB on the HDAC8 mRNA expression in CRC cell lines

Two human colorectal cancer cell lines, HT-29 and HCT-116, were utilized. We had previously investigated the effect of NaB on the proliferation of the two cell lines by using MTT assay after 24, 48 and 72 hours of treatment (Ghiaghi et al., 2019[15]). According to our previous study, we showed that NaB inhibited the growth of the two cell lines in a dose- and time-dependent manner. The IC_50_ values for the HT-29 cell line were 65, 18.6, and 9.2 mM after 24, 48, and 72 hours of treatment, respectively. The IC_50_ values for the HCT-116 cell line were 35.5, 9.6, and 10 mM after 24, 48, and 72 hours of treatment, respectively (Ghiaghi et al., 2019[15]). 

To determine the effect of NaB on the mRNA expression of HDAC8 in two human colorectal cancer cell lines (HT-29 and HCT-116), we treated cell lines with different concentrations of NaB based on IC_50_ for 24 and 48 hours and subsequently, mRNA expression was determined by Real-Time PCR.

**Figure 1:**
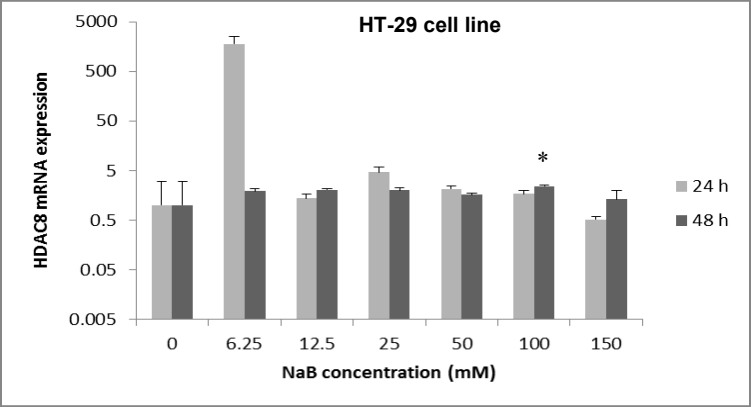
The effect of sodium butyrate (NaB) on HDAC8 mRNA expression on the HT-29 cell line. Cell lines were seeded (50×10^3^ cells/well in 96-well plates) and were treated for 24 hours and 48 hours with 6.25 mM to 150 mM concentrations of NaB at triplicate experiments and compared with the control group. Untreated cells (0 mM) served as the control group. The levels of HDAC8 mRNA were determined by qRT-PCR using the comparative ΔΔCt method of quantification. Results were normalized to GAPDH. P-value <0.05 was considered to be statistically significant. Error bars represent mean ± S.D. * Indicates a significant decrease (p<0.05).

Both cell lines (HT-29 and HCT-116) were treated for 24 and 48 hours with 6.25 mM to 150 mM of NaB. The HDAC8 mRNA expression in treated HT-29 cells with 150 mM of NaB for 24 hours was significantly decreased compared to the control group (untreated cells) (p<0.05) (Figure 1(Fig. 1)). It was found that NaB reduced the expression of HDAC8 mRNA by 1.75 fold in comparison with the control group (0 mM). In contrast, the treatment of HT-29 cells with different concentrations of NaB for 48 hours, showed that the mRNA expression of HDAC8 was increased at 150 mM of NaB, but this was not significant in comparison with the control group (0 mM) (p>0.05) (Figure 1(Fig. 1)). As shown in Figure 1(Fig. 1), NaB caused a dose- and time-dependent reduction of HDAC8 mRNA expression.

**Figure 2:**
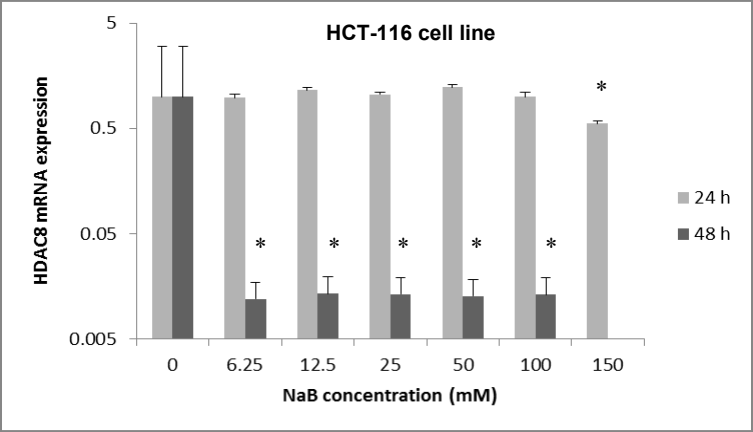
The effect of sodium butyrate (NaB) on HDAC8 mRNA expression on the HCT-116 cell line. Cell lines were seeded (50×10^3^ cells/well in 96-well plates) and were treated for 24 hours with 6.25 mM to 150 mM concentrations of NaB and they were treated for 48 hours with 6.25 mM to 100 mM concentrations of NaB at triplicate experiments and compared with the control group. Untreated cells (0 mM) served as the control group. The levels of HDAC8 mRNA were determined by qRT-PCR using the comparative 2^-ΔΔCt^ method of quantification. Results were normalized to GAPDH. P-value <0.05 was considered to be statistically significant. Error bars represent mean ± S.D. * Indicates a significant decrease (p<0.05).

Also, the HDAC8 mRNA expression in treated HCT-116 cells with 150 mM of NaB for 24 hours was significantly decreased in comparison with the control group (untreated cells) (p<0.05) (Figure 2(Fig. 2)). In contrast, the HDAC8 mRNA expression in HCT-116 cells treated with all concentrations of NaB (6.25 mM to 100 mM) for 48 hours was significantly decreased in comparison with the control group (0 mM) (p<0.05) (Figure 2(Fig. 2)). 

### Molecular docking study

**Figure 3:**
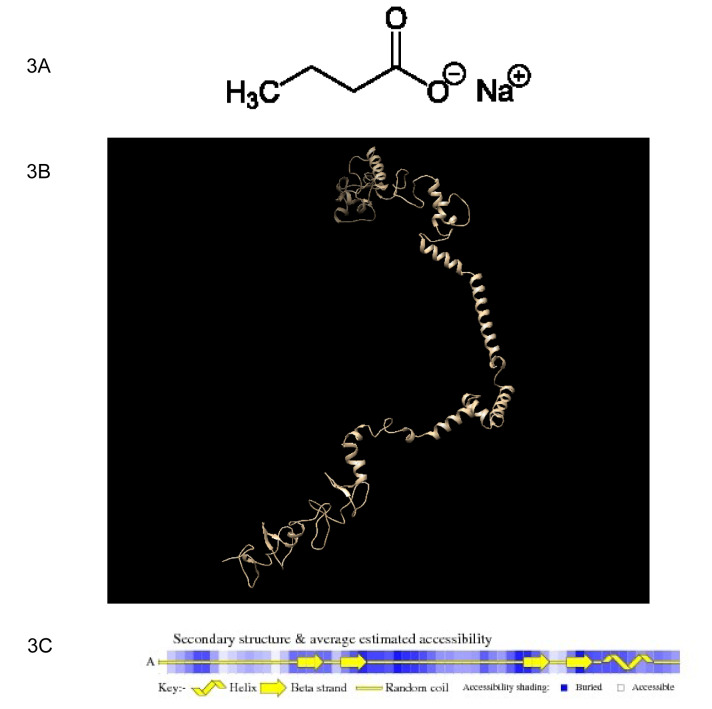
The structure of sodium butyrate (NaB) and LHX1. A) Structure of NaB. B) Display of the predicted structure of the LHX1 protein by Chimera software. C) Propagation of the secondary structure of the LHX1 protein by VADAR. H: Helices, β: Beta strand, C: Random coil.

LHX1 is one of the transcription factors of the HDAC8 gene that plays an important role in gene regulation, determining the life span of the cell (Phillips, 2002[37]; Karavanov et al., 1998[24]; Kobayashi et al., 2004[27]; Ye et al., 2012[56]). Tertiary structure prediction and docking studies of the protein were performed to understand the role of this protein and its interactions with NaB. PDB LHX1 protein was available in the database. Therefore, using the PDB of this protein and I-tasser and Galaxy refine servers, the 3D molecular structure was predicted, and using the Modeller software (version 17), the best model with the lowest energy levels and DOPE score for atoms was selected. Then the 3D protein structure was presented by the Chimera software (version 1.11.2). The structure of NaB and LHX1 is shown in Figures 3A and B(Fig. 3). On the other hand, the propagation of the secondary structure of the LHX1 protein was done using the VADAR server (Figure 3C(Fig. 3)).

**Figure 4:**
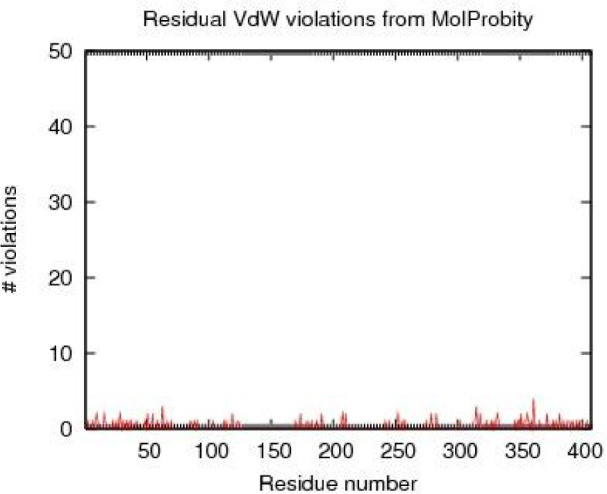
The MolProbity diagram of the LHX1 protein, which confirmed the quality of the model.

The RMS deviation for covalent bonds relative to the standard dictionary was 0.019 Angstroms. Every covalent bond lied within a 6.0*RMSD limit about the standard dictionary values. Additionally, the MolProbity chart showed hydrogen bands and close Vandervelde contacts. According to the results, most of the amino acids in the LHX1 protein had an acceptable position in terms of hydrogen bonding and Vandervelde bonds (Figure 4(Fig. 4)).

**Figure 5:**
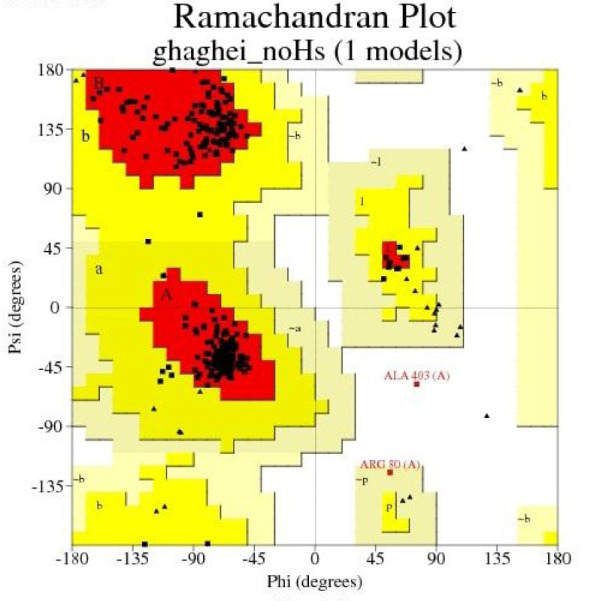
The Ramachandran contour plot of LHX1 depicting Φ and Ψ angles. The most desirable regions are colored red, additionally allowed, generously allowed and disallowed regions are indicated as yellow, light yellow and white fields respectively.

Results from the PROCHECK analysis of LHX1 are given in Table 2(Tab. 2), and the Ramachandran plot generated by the same program is represented in Figure 5(Fig. 5). 

**Figure 6:**
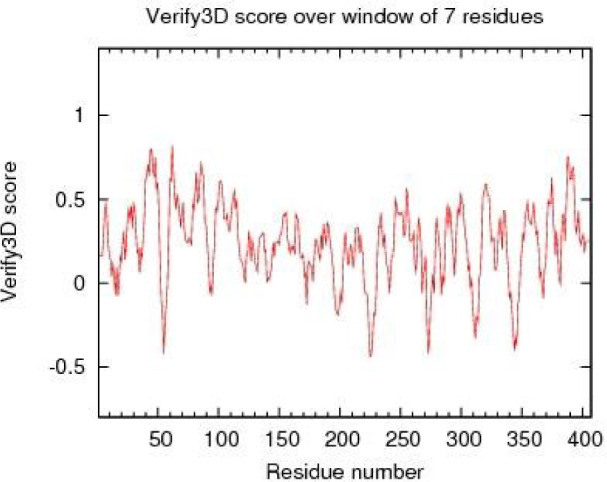
The Verify 3D diagram of the LHX1 protein, which confirmed the quality of the model.

Verify 3D profile for the final structure of LHX1 is shown in Figure 6(Fig. 6) which explains that most of the residues have a score over 0.2 (residues with a score over 0.2 are reliable).

To perform molecular docking with NaB, it was necessary to find amino acid binding sites in the LHX1, for which purpose, the 3D ligand site and the Uchicago.edu/binding sites were used. After reviewing the sites, the best binding residues of LHX1 were selected with their respective amino acids (first, second, and third connection positions), which are shown in Table 3(Tab. 3).

**Figure 7:**
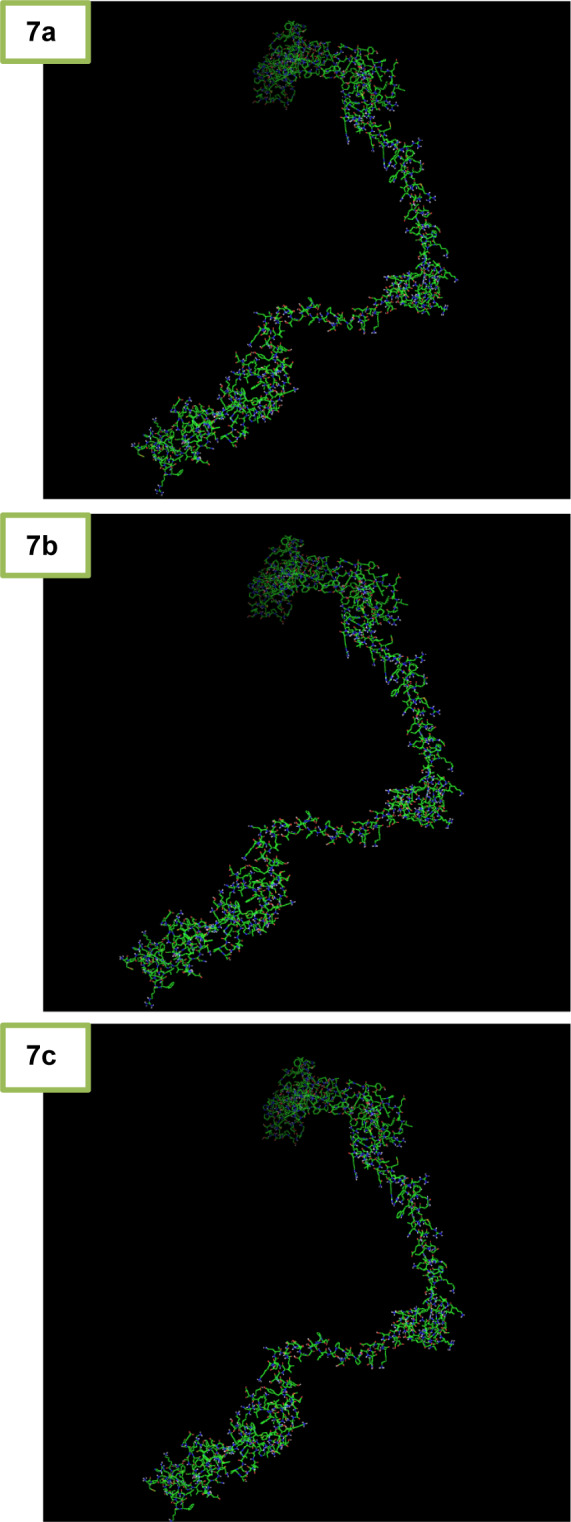
Different modes of Docking and the connection between LHX1 and sodium butyrate (NaB) are shown in the Pymol software. a) Binding residues 1, the amino acid participating in the joint, including G182, P183, R184, T185, I187, V223, V226, W227, and N230. b) Binding residues 2, the amino acid participating in the joint, including R210, Q225, Q229, and R232. c) Binding residues 3, the amino acid participating in the joint, including K204, Q229, and R232.

The Autodock Vina software (version 4.2.6) was used for analysis of the docking and connection between LHX1 and NaB. Each of the LHX1 attachment sites with the amino acids mentioned in Table 2(Tab. 2) was separately imported into the Autodock vina software, and the application was allowed to search all the dimensions of the protein. At this stage, after performing 100 docking operations, the site with the lowest binding energy between LHX1 and NaB was selected. Then the Pymol software (version 1.7.4.5 Edu) was used to display the connection between LHX1 and NaB (Figure 7(Fig. 7)). 

**Figure 8:**
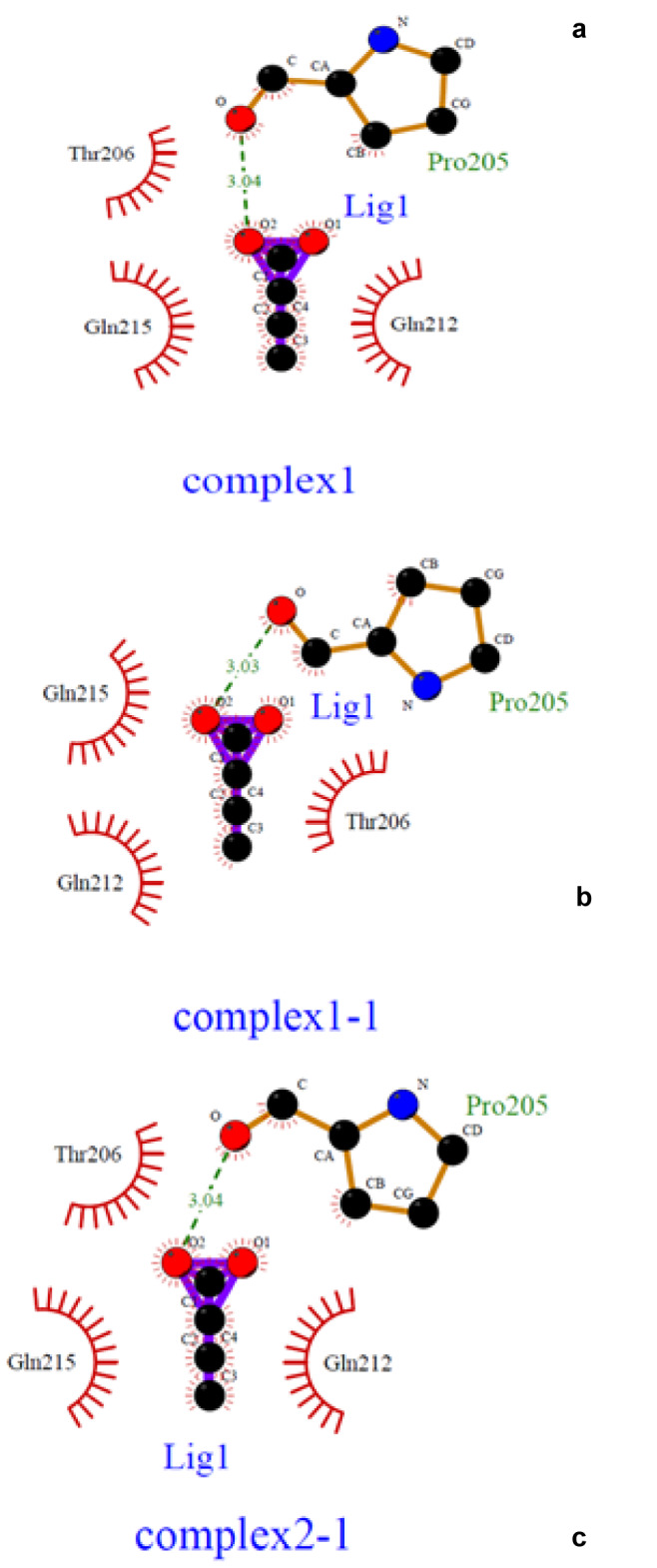
Different models of Docking and the connection between LHX1 and sodium butyrate (NaB) are shown by the Ligplot software. a) Binding residues 1, the amino acid participating in the joint, including G182, P183, R184, T185, I187, V223, V226, W227, and N230. b) Binding residues 2, the amino acid participating in the joint, including R210, Q225, Q229, and R232. c) Binding residues 3, the amino acid participating in the joint, including K204, Q229, and R232. The multipoint line represents the hydrogen bond. Solar states indicate non-polar bonds.

Finally, using the Ligplot software (version 4.5.3) the 2D structure of the protein-ligand complex was obtained (Figure 8(Fig. 8)). Among the 3 complexes obtained, the complex 1-1 (Figure 8B(Fig. 8)), was acceptable which contained the amino acids R210, Q225, Q229, and R232, because the length of the hydrogen bond was 3.03 nM, which was smaller than two other complexes and it was postulated that the shorter the length of the bond, the more acceptable the outcomes. The model comprising of the most desirable scores was selected as the theoretical model for LHX1 protein.

## Discussion

Histone acetylation plays an important role in the remodeling of chromatin and regulation of gene transcription (Johnstone, 2002[23]; Iizuka and Smith, 2003[22]). Lysine acetylation is controlled by the counteracting function of two types of enzymes namely, histone acetylase (HAT) and histone deacetylase (HDAC) enzymes (Bannister and Kouzarides, 2011[3]). Imbalances between the activities of HATs and HDACs are associated with some diseases including cancers (Singh et al., 2018[41]). In such pathological conditions, classical HDACs have shown overexpression. It appears that the inhibition of the HDAC ezyme could potentially prevent such diseases; therefore, histone-deacetylase inhibitors (HDACis) have emerged as promising agents for cancer treatment (Fraga et al., 2005[14]; Khan and La Thangue, 2012[25]). One of these HDACis is sodium butyrate (NaB) (Emenaker et al., 2001[10]; Hinnebusch et al., 2002[20]). In a study published in 2014 Tailor et al. examined the effects of NaB on the induction of apoptosis in the HCT-116 human colorectal cancer cell line, and observed that treatment with a concentration of 1-5 mM of NaB, administered for 12 and 24 hours resulted in decreased cell survival, cell cycle G2-M phase arrest and increased apoptosis in the HCT-116 cells line (Tailor et al., 2014[45]). Furthermore, by using an MTT assay in present study, it was examined that NaB showed cytotoxicity on HT-29 and HCT-116 colorectal cancer cell lines. Namely, in the HT-29 cell line, the percentage of cell viability was 52 %, 52 %, and 50 % after 24, 48 and 72 hours, respectively. Also, in the HCT-116 cell line, the percentage of cell viability was 68 %, 51 %, and 54 % after 24, 48 and 72 hours, respectively. Our results further showed that NaB-induced cell death occurred in a dose- and time-dependent manner.

Additionally, NaB has been shown to play a role in inhibiting histone deacetylases and had the highest inhibitory effect on HDAC8 (Cummings and Macfarlane, 1991[9]).This substance also seemed to be produced naturally in the gastrointestinal tract, therefore, it did not cause side effects when used as a medical substance in the treatment of diseases (Butler et al., 2001[8]; He et al., 2001[18]). NaB also displayed inhibitory properties such as cell cycle stopping, suppression of cell differentiation, and induction of cell apoptosis (Nakano et al., 1997[35]). In this study, we used the human colorectal carcinoma HCT-116 and the colorectal adenocarcinoma HT-29 cell lines, in order to investigate the effects of NaB on HDAC8 gene expression. Previously, no studies had been conducted on the effects of NaB on HDAC8 gene expression. In 2013, Yan et al. examined the effects of NaB and SAHA as histone deacetylase inhibitors in suppressing P53 mutant cells via HDAC8 in the cell lines of SW480 and HacaT. They observed that HDAC inhibitors reduced the transcriptional level of wild-type P53 genes via HDAC8 (Yan et al., 2013[55]). Also, in 2016, Singh et al. examined the effects of Silymarin, NaB and TSA on the expression of class I HDACs, ZEB1, and E-cadherin in the migration of lung cancer cells. In their study, A549 and H460 lung cancer cells were treated with NaB and TSA for 24 hours separately. They concluded that NaB decreased expression of HDAC1 and HDAC2 (P<0.05) while increasing the expression of E-cadherin. NaB also interrupted the binding of HDAC1 and HDAC2 to ZEB1 in lung cells (Singh et al., 2016[42]). In the present study, we also investigated the effects of NaB on HDAC8 mRNA expression in two HCT-116 and HT-29 human colorectal cancer cell lines. Our results showed that in HCT-116 and HT-29 cell lines, NaB at a 150 mM concentration significantly caused dose- and time-dependent reduction of mRNA HDA in the HTC-116 cell line, and NaB at 6.25 mM to 150 mM concentrations caused significant reduction in HDAC8 mRNA expression levels (p<0.05). These findings provided evidence that NaB inhibited the growth and proliferation by affecting the expression of histone deacetylase genes. Vannini et al. showed that knockdown of HDAC8 using siRNA inhibited human cervical, colon, and lung cancer proliferation (Vannini et al., 2004[50]).

Furthermore, HDACis were shown to be effective anti-cancer agents, exhibiting antineoplastic properties through inhibition of migration and invasion, cell cycle arrest, and the induction of apoptosis in several cancer cells (Gumbarewicz et al., 2016[17]; Grabarska et al., 2017[16]). In previous research, NaB affected gene expression through binding to transcription factors. In addition, HDAC8 played a role in controlling the morphogenesis of the skull, and performed its epigenetic action by suppressing Otx2 and LHX1 (Saha et al., 2013[39]). Moreover, in order to better understand the molecular mechanism of NaB action and to identify LHX1 as the transcription factor of HDAC8 interacting with NaB, we performed the docking study between NaB and LHX1. The important role of the transcription factor was important, since, LHX1 was blocked by NaB and subsequently, the transcription from the HDAC8 gene was decreased which resulted in cell-cycle arrest and cell death. In a previous publication, Tatar et al. conducted docking between NaB and HDAC8, and the results showed that there was a connection between NaB and HDAC8 (Tatar et al., 2011[46]). Our results further showed that NaB was strongly bound to LHX1, and probably competitively inhibited the protein binding of LHX1 to HDAC8. In our work, the most desirable structure of LHX1 was selected with the lowest energy level. Using the Ramachandran diagrams, MolProbity and Verify3D it was concluded that the structure chosen was a high-quality model. Using the sites of the 3D ligand site, Uchicago.edu/binding site and Interproscan were selected as the best linking locations in the LHX1. By using the Autodock vina software the connection between LHX1 and NaB in a place with the least energy connection was established. The two-dimensional structure of the protein-ligand complex was obtained with the Ligplot software. Our results demonstrated the complexity of the amino acids R210, Q225, Q229 and R232 in the LHX1 structure and showed that they had a more integrated connection with NaB because the length of the hydrogen bond was 3.03, which was a smaller bond length than the other complexes. 

## Conclusion

Finally, the results of this study showed that NaB could downregulate HDAC8 expression to inhibit the growth of HT-29 and HCT-116 human colorectal cancer cell lines and promote cell apoptosis. Our *in silico* study provided valuable information about the structural analysis of NaB/LHX1 interaction and revealed the important residues of LHX1 involved in the interaction with NaB. These results suggested that NaB could strongly bind to the transcription factor of HDAC8 and subsequently decrease the expression of the HDAC8 gene in cancer cells. This may also be one of the molecular mechanisms underlying the manner by which NaB induced human colorectal cancer cell arrest and exerted its anti-tumor effects on such cells. Further studies are probably needed in the future to evaluate other likely mechanisms of action of NaB on the inhibition of human cancer cell lines.

## Funding sources

No funding sources.

## Conflict of interest

None declared.

## Figures and Tables

**Table 1:**
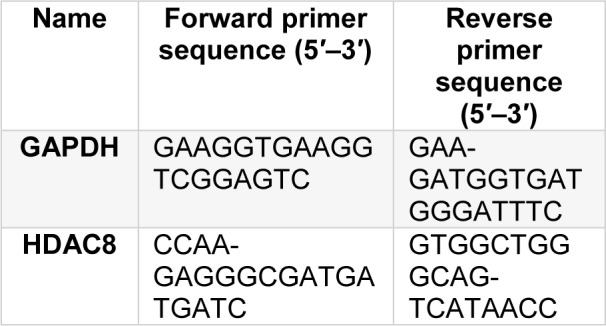
Primer sequences for qRT-PCR

**Table 2:**
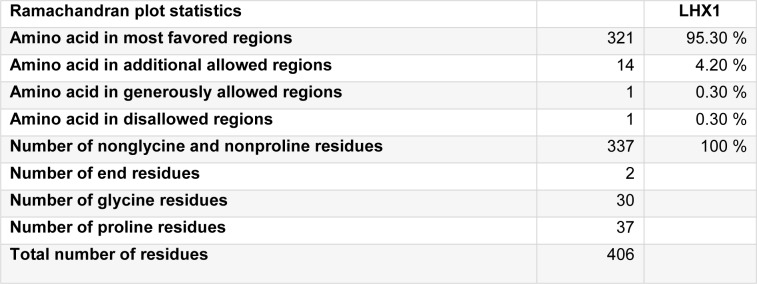
Statistics of the 3D model of LHX1 from the Ramachandran plot

**Table 3:**
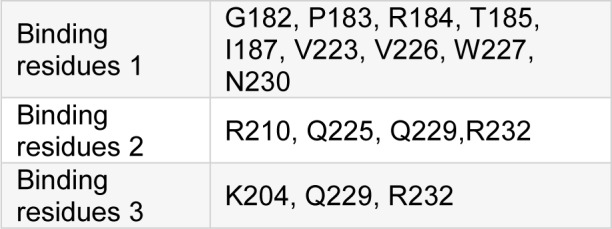
Binding residues of LHX1 protein
